# First- and Second-Line Targeted Systemic Therapy in Hepatocellular Carcinoma—An Update on Patient Selection and Response Evaluation

**DOI:** 10.3390/diagnostics6040044

**Published:** 2016-11-28

**Authors:** Johann von Felden, Kornelius Schulze, Ines Gil-Ibanez, Tobias Werner, Henning Wege

**Affiliations:** I. Department of Internal Medicine, University Medical Center Hamburg-Eppendorf, Martinistraße 52, 20246 Hamburg, Germany; j.von-felden@uke.de (J.v.F.); k.schulze@uke.de (K.S.); i.gil-ibanez@uke.de (I.G.-I.); T.werner@uke.de (T.W.)

**Keywords:** hepatocellular carcinoma, targeted systemic therapy, sorafenib, regorafenib, prediction of treatment response, mRECIST

## Abstract

Advanced hepatocellular carcinoma (HCC) with vascular invasion and/or extrahepatic spread and preserved liver function, according to stage C of the Barcelona Clinic Liver Cancer (BCLC) classification, has a dismal prognosis. The multi-targeted tyrosine-kinase receptor inhibitor (TKI) sorafenib is the only proven active substance in systemic HCC therapy for first-line treatment. In this review, we summarize current aspects in patient selection and management of side effects, and provide an update on response evaluation during first-line sorafenib therapy. Since second-line treatment options have been improved with the successful completion of the RESORCE trial, demonstrating a survival benefit for second-line treatment with the TKI regorafenib, response monitoring during first-line therapy will be critical to deliver optimal systemic therapy in HCC. To this regard, specific side effects, in particular worsening of arterial hypertension and diarrhea, might suggest treatment response during first-line sorafenib therapy; however, clear predictive clinical markers, as well as laboratory test or serum markers, are not established. Assessment of radiologic response according to the modified Response Evaluation Criteria in Solid Tumors (mRECIST) is helpful to identify patients who do not benefit from sorafenib treatment.

## 1. Introduction

Despite milestone achievements in targeted systemic therapy, hepatocellular carcinoma (HCC) is still one of the deadliest cancers worldwide. Due to the spread of hepatitis C, incidence rates are rising in Western countries. Unfortunately, even in developed regions, in the majority of patients HCC is diagnosed at an advanced stage without curative treatment options. Since the successful SHARP trial in 2008 and, most recently, the RESORCE trial, the multi-targeted receptor tyrosine-kinase inhibitors (TKI) sorafenib and regorafenib are proven to be active substances in systemic HCC therapy. In this review, we will summarize current indications and patient selection for targeted systemic therapy in HCC. We will highlight treatment guidance employing clinical parameters, biomarkers, and imaging, and provide an update on treatment options after the successful RESORCE trial for second-line treatment with regorafenib, as well as novel treatment options currently under evaluation in clinical trials.

## 2. Indications for Targeted Systemic Therapy with Sorafenib in Hepatocellular Cancer

In HCC, curative treatment options are available for patients classified as early stage (stage 0 or A) in the Barcelona Clinic Liver Cancer (BCLC) staging system [1]. Palliative transarterial chemoembolization (TACE) is the standard of care for patients with liver-limited disease and without portal vein thrombosis (BCLC stage B). Targeted systemic therapy with the TKI sorafenib is the first-line systemic treatment for advanced HCC in patients with preserved liver function (BCLC stage C). In 2008, the SHARP trial (A Phase 3 Study of Sorafenib in Patients With Advanced HCC, clinicaltrials.gov registry number [NCT]: NCT00105443) demonstrated a prolonged median overall survival (OS) of 10.7 months in the sorafenib arm vs. 7.9 months (hazard ratio (HR) 0.69, 95% confidence interval (CI) 0.55–0.87, *p* < 0.001) in the placebo arm [2]. Selected clinical trials investigating systemic therapy in HCC are summarized in Table 1. Consecutive sub-group analyses revealed safe and effective treatment with sorafenib independent of alanine aminotransferase (ALT), aspartate aminotransferase (AST), alpha-fetoprotein (AFP), and bilirubin serum levels [3]. Moreover, liver function remained stable during therapy with sorafenib [3]. An additional substudy verified a consistent benefit for sorafenib regarding median OS in patients with advanced-stage disease, irrespective of the underlying risk-factor, extent of the tumor burden, clinical performance status, and prior treatment [4]. Interestingly, in this substudy, the subset of patients with hepatitis C–associated HCC had a better response to sorafenib and a superior median OS compared to the placebo than patients with non-hepatitis-C–associated HCC (14.0 vs. 7.4 months, HR 0.50, 95% CI 0.32–0.77). Whereas the SHARP study primarily included patients from Europe and America, a second pivotal trial enrolled Asian-Pacific patients and demonstrated an equal overall treatment benefit (median OS 6.5 vs. 4.2 months, HR 0.68, 95% CI 0.50–0.93, *p* = 0.014) despite several discrepancies in baseline characteristics [5]. Combining these two major phase 3 trials, extensive clinical data established a robust sorafenib-driven benefit in median OS independent of the underlying HCC-causing risk factor profile [6,7]. Since 2008, many additional real-life reports have confirmed the benefit of sorafenib treatment in patients with HCC, above all the global, prospective, non-interventional GIDEON trial (Global Investigation of Therapeutic Decisions in HCC and of Its Treatment With Sorafenib, NCT00812175) with more than 3000 patients worldwide [8].

Since sorafenib has a proven benefit in advanced-stage HCC in patients with preserved liver function, it was anticipated to expand its role to intermediate-staged HCC in terms of a combinational treatment approach in BCLC stage B or as an adjuvant treatment following curative therapy. Within the SPACE trial (A Phase 2 Randomized, Double-blind, Placebo-controlled Study of Sorafenib or Placebo in Combination With TACE Performed With DC Bead and Doxorubicin for Intermediate Stage HCC, NCT00855218), Lencioni and colleagues investigated the impact of sorafenib in patients with intermediate-stage multinodular HCC. The patients in the trial received TACE with doxorubicin-eluting beats (DEB) in combination with sorafenib or placebo [9]. In spite of the practical feasibility and manageable toxicities of DEB-TACE in combination with sorafenib treatment, the exploratory phase 2 trial was ultimately not able to demonstrate a benefit in the primary endpoint time-to-tumor progression (TTP) and the secondary endpoint median OS [9]. A phase 3 clinical trial, focusing on Japanese and Korean patients with intermediate-stage HCC, investigated the benefit of sorafenib after TACE [10]. In this study, the primary and secondary endpoints TTP and OS were also not reached in this mainly Hepatitis C Virus (HCV)-associated HCC patient cohort [10]. Recently presented, a study including 294 patients from 20 sites in the United Kingdom and investigating a combination of DEB-TACE and sorafenib or placebo again revealed no benefit of the combination therapy regarding median progression-free survival (PFS) as a primary endpoint (7.8 vs. 7.7 months, HR 1.03, 95% CI 0.75–1.42, *p* = 0.85) [11].

In a different therapeutic setting, the phase 3, randomized, placebo-controlled, double-blind STORM trial (Sorafenib as Adjuvant Treatment in the Prevention Of Recurrence of HCC, NCT00692770) investigated the benefit of sorafenib following resection or local ablation [12]. Unfortunately, this multinational study also failed to achieve its primary endpoint of a significant benefit in median recurrence-free survival (RFS) between patients given sorafenib and placebo (33.3 vs. 33.7 months, HR 0.94, 95% CI 0.78–1.134, *p* = 0.26) [12]. Notably, the standard dose of sorafenib was intolerable in a high frequency of HCC patients enrolled in the STORM trial [12]. In this regard, another study demonstrated a significant association of hepatic dysfunction and sorafenib intolerability [13].

In summary, sorafenib is well established as the first-line, systemic, targeted HCC therapy in patients with preserved liver function. Based on the available data, sorafenib is not recommended as an adjuvant therapy in patients after complete tumor removal or destruction and most likely has no benefit in the majority of patients receiving palliative TACE. The disappointing results of the above-mentioned trials underline the importance of further investigations in HCC-directed treatment strategies in addition to sorafenib.

## 3. Common Adverse Events of Treatment with Sorafenib

The most frequent side effects of sorafenib treatment are reported to be dermatological reactions, mainly hand-foot syndrome (HFS), fatigue, diarrhea, and arterial hypertension. Besides dose reduction or interruption of treatment, complementary symptomatic regimens are required for these adverse events. For the HFS, topical urea, avoidance of hot water, moisturizing lotions, and salicylate creams are useful options [14]. Regarding fatigue, underlying co-morbidities such as cirrhosis, hypothyroidism, anemia, and depression are the targets of supportive treatment approaches [14]. Loperamide is the choice of symptomatic treatment in cases of sorafenib-associated diarrhea [14]. Although most patients with HCC suffer from liver cirrhosis and systemic hypotension, some patients might present with arterial hypertension after initiating sorafenib. With coexisting varices, a non-selective beta-blocker is the first choice; otherwise sartans or hydrochlorothiazide should be preferred. However, dose reductions or treatment interruptions of sorafenib are frequently required.

## 4. Biomarkers for Response to Targeted Systemic Therapy with Sorafenib

It is crucial to understand that a variable can influence the outcome of the total population suffering from the disease, but it may not predict the response to treatment, as its influence may be regardless of a given treatment. These variables mostly reflect the severity of the disease itself. On the other hand, a variable has a true predictive value for treatment response if it stratifies into better and worse outcome within the treatment group itself.

In a subset analysis of patients enrolled in the SHARP trial, Raoul et al. analyzed the influence of elevated aminotransferases, AFP, and bilirubin on the outcome of sorafenib treatment. Patients with elevated levels of these parameters had a better median OS with sorafenib compared to the placebo group, but had a worse median OS compared to those patients with non-elevated levels of these markers at baseline, irrespective of treatment with sorafenib or placebo. The authors concluded that these markers had a prognostic value in terms of disease severity, but had no predictive value for the effectiveness of sorafenib treatment in patients with advanced HCC [3]. In a Korean population, Lee and colleagues described that Child-Pugh class A, tumor diameter <5 cm, low baseline AFP levels, and the occurrence of skin toxicity (HFS) with grade ≥2 were independent favorable predictive factors in sorafenib-treated patients for both median OS (6.0 vs. 2.8, 6.0 vs. 4.3, 5.8 vs. 4.1, and 5.9 vs. 4.0 months, respectively, all *p* < 0.05) and PFS (4.3 vs. 2.1, 3.9 vs. 2.8, 5.6 vs. 2.8, and 4.5 vs 2.6 months, respectively, all *p* < 0.05) [15]. Personeni et al. investigated the dynamics of AFP levels under treatment with sorafenib. They defined a 20% decrease of initially elevated AFP after eight weeks of treatment as the AFP response and showed a correlation with a higher median OS (13.3 vs. 8.2 months, *p* < 0.05) and TTP (7.9 vs. 2.4 months, *p* < 0.01) in treated patients [16]. 

There has been a controversial discussion regarding if the occurrence of common side effects such as arterial hypertension, diarrhea, or HFS may have predictive value for the treatment response to sorafenib. The development of hypertension as an adverse event under treatment with sorafenib was correlated with a significantly longer median OS (18.2 vs. 4.5 months, *p* = 0.016) in treated patients with Child-Pugh class A and B cirrhosis [17]. Along this line, Bettinger et al. showed in their cohort of 112 HCC patients treated with sorafenib that the occurrence of diarrhea might serve as a predictive marker for a treatment response with a prolonged median OS (14.1 vs. 7.1 months, HR 0.41, *p* = 0.011). In their cohort, HFS was not associated with OS or TTP, but BCLC stage was a negative independent prognostic factor [18]. In this regard, Ponziani et al. found that adjustment of the sorafenib dose because of relevant side effects, to induce tolerability rather than stopping the drug, prolonged OS and TTP, and achieved even better results compared to the treatment group with minor side effects and unchanged treatment with 800 mg per day (OS 12.5 vs. 5.7 months, HR = 0.4, *p* < 0.0001, and TTP 9.5 vs. 3 months, HR = 0.3, *p* < 0.0001) [19]. A Japanese group performed a retrospective, propensity score matching analysis to investigate the effect of starting the treatment with an initial dosage of 400 mg per day vs. the recommended 800 mg per day (including the option to adjust the dose depending on tolerance and side effects). They found no differences in median OS (9.2 vs. 9.7 months, *p* = 0.350), PFS (3.4 vs. 3.2 months, *p* = 0.729) and disease control rate (*p* = 0.719). Surprisingly, grade 3 or more severe adverse events (SAE) were distributed equally between the two groups (26.6% vs. 23.7%, *p* = 0.580) [20].

Regarding experimental biomarkers, a subanalysis of the SHARP cohort showed a prognostic value of vascular endothelial growth factor (VEGF) and angiotensin 2 (Ang2) in patients with advanced HCC concerning median OS regardless of treatment with sorafenib or placebo, while a significant role in predicting the response to sorafenib treatment could not be shown [21]. Alterations in the fibroblast growth factor (FGF) and the corresponding receptor (FGFR) were also proposed to influence the response to sorafenib by Arao et al. However, due to a very small cohort of patients, mostly with viral hepatitis as the underlying liver disease, their findings are limited [22,23]. Vaira et al. investigated the predictive value of micro-RNA expression within HCC. They found that higher levels of miR-425-3p predict longer TTP and PFS (HR = 0.4, *p* = 0.0008, and HR = 0.5, *p* = 0.007, respectively), and are associated with cell death and reduced cell motility in vitro [24]. Recently, Lee et al. performed a genome-wide association study (GWAS) in HCC patients treated with sorafenib and found that patients with a genetic variation in the SLC15A2 gene showed a longer PFS (HR = 2.18, *p* = 0.003). The authors suggest a role of SLC15A2 in the sorafenib metabolism [25].

In summary, there is still no established laboratory test to reliably predict response to sorafenib treatment in advanced HCC. However, side effects due to sorafenib, in particular arterial hypertension and diarrhea, might suggest a benefit of treatment. Strategies to adjust dosage and to manage tolerability, rather than stopping targeted treatment, have been proposed. Unfortunately, most publications dealing with parameters to predict response to sorafenib treatment were retrospective. This severely limits the translational relevance of these data, leaving an urgent need to convincingly outline clinical or molecular biomarkers to predict response to sorafenib and thus to allow individualized treatment and improve outcomes of advanced HCC in the future.

## 5. Imaging to Predict Response to Targeted Systemic Therapy with Sorafenib

Already in 2000, the consensus conference of the European Association for the Study of the Liver (EASL) on HCC stated that the level of vascularization of HCC is the key hallmark to identify the response to targeted systemic therapy. This led to an HCC-specific revision of the Response Evaluation Criteria in Solid Tumors (RECIST) [26]. The modified RECIST (mRECIST), briefly summarized in Table 2, are based on multiphase computed tomography scans and focus not on the whole tumor mass but on the contrast-enhanced portion of hepatic lesions [27]. Objective response (OR) vs. stable disease (SD) or progressive disease (PD) analyzed by mRECIST was significantly correlated with OS and predicted OS under treatment with sorafenib (18.2 vs. 7.7 months), while RECIST was not able to distinguish these patients [28]. Similar results were also seen in a retrospective subanalysis of patients with advanced HCC treated with brivanib vs. placebo. PD assessed by mRECIST was associated with a shorter OS compared to PD assessed by World Health Organization (WHO) criteria [29]. Recently, Zocco and co-workers analyzed the role of contrast-enhanced ultrasound (CEUS). Short-term changes in the perfusion characteristics 15 days after starting treatment with sorafenib predicted OS and PFS [30]. In a small cohort, also 18F-FDG positron emission tomography (PET) was able to predict OS and PFS in patients treated with sorafenib depending on the standardized uptake value (SUV) of FDG [31]. Significant changes of parameters in magnetic resonance imaging (MRI) under sorafenib treatment have also been described, but unfortunately do not correlate to treatment response [32,33]. It is of note that MRI is the most accurate and sensitive liver imaging technique, especially regarding the detection and staging of small intrahepatic nodules <2 cm [34]. 

In summary, CEUS has the potential to predict the response to sorafenib treatment early, while mRECIST in CT scans is currently the recommended and most commonly employed imaging technique to monitor treatment response.

## 6. Alternative First-Line Treatment Options

Since the positive SHARP trial in 2008, no other systemic agent had a proven benefit for first-line treatment in systemic HCC. Lately, immune-mediated anticancer therapy with checkpoint inhibitors, in particular nivolumab, pembrolizumab, and ipilimumab, has had a great impact on several advanced cancer etiologies, e.g., melanoma or lung cancer [35,36]. In advanced HCC, there are some studies investigating the effect both in first- and second-line treatment. The CheckMate 459 study (A Study of Nivolumab Compared to Sorafenib as a Primary Treatment in Patients With Advanced HCC, NCT02576509) is an ongoing, open-label, phase 3 study to investigate the effect of the PD-1 inhibitor nivolumab vs. sorafenib as a first-line treatment in advanced HCC. Supporting the anticancer activity of the immune system is also the goal of trials investigating the effect of oncolytic viruses. The PHOCUS study (HCC Study Comparing Vaccinia Virus Based Immunotherapy Plus Sorafenib vs. Sorafenib Alone, NCT02562755), is an ongoing, open-label, phase 3 trial comparing the OS of vaccinia virus based immunotherapy followed by sorafenib vs. sorafenib alone.

## 7. Second-Line Treatment Options

Assessment of a negative treatment response to sorafenib ultimately leads to the question of an alternative or second-line treatment. Just recently reported, the RESORCE trial (Study of Regorafenib After Sorafenib in Patients With HCC, NCT01774344) demonstrated a significant improvement in median OS for patients treated with regorafenib vs. placebo as a second-line treatment after radiologic progression under sorafenib (10.6 vs. 7.8 months, HR = 0.62, 95% CI 0.50–0.78, *p* < 0.001) [37]. This is the first positive trial besides the SHARP trial after almost a decade of negative trials for systemic treatment for HCC. There are several reasons controversially discussed to be responsible for the failure of first- and second-line studies in the past. The main ones are (i) heterogeneity of study populations and the lack of patient selection according to molecular signatures; and (ii) toxicity concerns vs. little antitumoral potency of experimental agents. The growing understanding of the molecular heterogeneity of HCC and its driving mutations in tumor evolution might be the key to conducting successful trials in the future [38]. 

The BRISK PS trial (Comparison of Brivanib and BSC to Placebo for Treatment of Liver Cancer for Those Subjects Who Have Failed Sorafenib Treatment, NCT00825955) for HCC patients with intolerance or progression under sorafenib showed a longer TTP for patients treated with brivanib vs. placebo (4.2 vs. 2.7 months, HR = 0.56, *p* < 0.001), but failed to show a significant benefit in the primary endpoint median OS (9.4 vs. 8.2 months, HR = 0.89, *p* = 0.3307) [39]. The EVOLVE-1 study (Global Study Looking at the Combination of RAD001 Plus BSC and Placebo Plus BSC to Treat Patients With Advanced HCC, NCT01035229), which investigated the effect of everolimus as a second-line treatment for patients intolerant to sorafenib or with progression under sorafenib, also remained negative (median OS was 7.6 vs. 7.3 months, HR = 1.05, *p* = 0.68) [40]. In contrast to the BRISK PS and EVOLVE-1 trials, the RESORCE trial included only patients with HCC progression and not intolerance to sorafenib. Therefore, the need to find second-line treatment options for patients intolerant to sorafenib remains unmet.

Currently, there are several second-line studies ongoing or recently closed with data expected. The ReLive study (Efficacy and Safety Doxorubicin-Transdrug Study in Patients Suffering From Advanced HCC, NCT01655693) is an ongoing, open-label, phase 3 study investigating doxorubicin-transdrug as a second-line treatment in patients with advanced HCC and progression or intolerance to sorafenib compared to best standard of care. Other trials focus on promising subgroups and employ biomarker-based enrichment to stratify patients, e.g., the METIV trial (Study of Tivantinib in Subjects With Inoperable HCC Who Have Been Treated With One Prior Therapy, NCT01755767) comparing tivantinib vs. placebo in patients with high expression of cMET who had progression after prior systemic treatment. Based on the REACH trial (A Study of Ramucirumab Drug Product and BSC vs. Placebo and BSC as 2nd-Line Treatment in Participants With HCC After 1st-Line Therapy With Sorafenib, NCT01140347), the VEGF antibody ramucirumab is currently being tested in patients with elevated AFP. In the full REACH population, median OS was 9.2 months with ramucirumab compared with 7.6 months with placebo (HR 0.87, 95% CI 0.72–1.05, *p* = 0.14) [41]. The numerical difference of 1.6 months between the two arms was not significant. However, a predefined analysis in patients with elevated AFP revealed a treatment benefit in this subgroup (median OS for patients with baseline AFP > 400 ng/mL 7.8 vs. 4.2 months, HR 0.674, 95% CI 0.51–0.90, *p* = 0.006). Therefore, a second study, the REACH-2 trial (A Study of Ramucirumab vs. Placebo in Participants With HCC and Elevated Baseline Alpha-Fetoprotein, NCT02435433), is now evaluating ramucirumab in the more vulnerable population of HCC patients with elevated AFP and progressive disease under sorafenib or intolerance of sorafenib.

A second-line study investigating immune-mediated anticancer effects is the KEYNOTE-240 trial (Study of Pembrolizumab vs. BSC in Participants With Previously Systemically Treated Advanced HCC, NCT02702401), an ongoing, open-label, phase 3 trial for second-line treatment with Pembrolizumab in patients with progression on previous systemic treatment of advanced HCC. 

## 8. Conclusions

In summary, sorafenib remains the standard of care for first-line systemic therapy in advanced HCC with preserved liver function. Side effects, including fatigue, arterial hypertension, diarrhea and HFS, are common and require supportive symptomatic treatment to induce tolerance to sorafenib rather than drug withdrawal. The presence of arterial hypertension and diarrhea might suggest a treatment benefit, but clear predictive markers, including established laboratory tests or serum markers, are not reliable. Assessment of the radiologic response according to mRECIST is helpful to identify patients who do not benefit from sorafenib treatment. Until now, all first-line studies comparing sorafenib to alternative TKIs or novel agents remained negative, although some ongoing trials investigating immunotherapy are promising.

Just recently, a breakthrough was achieved in second-line treatment options for patients who progressed on sorafenib after almost a decade with negative studies. Regorafenib is the first systemic agent in patients with progression under sorafenib achieving a significant and meaningful benefit compared to a placebo. For patients not tolerating sorafenib, clinical trials still remain the only second-line therapeutic option.

## Figures and Tables

**Table 1:** Selected clinical trials investigating systemic therapy of HCC.

Name	Phase	Treatment Line	Study Drug	Primary Endpoint	Identifier
*Positive Trials*					
SHARP	3	First-line	Sorafenib vs. placebo	OS 10.7 vs. 7.9 months (HR 0.69, 95% CI 0.55–0.87, *p* < 0.001)	NCT00105443
RESORCE	3	Second-line	Regorafenib vs. placebo	OS 10.6 vs. 7.8 months (HR 0.62, 95% CI 0.50–0.78, *p* < 0.001)	NCT01774344
*Negative Trials*					
STORM	3	Adjuvant	Sorafenib vs. placebo	No difference in RFS (HR 0.94, 95% CI 0.78–1.13, *p* = 0.26)	NCT00692770
SPACE	2	First-line	DEB-TACE +/− sorafenib	No difference in TTP (HR 0.79, 95% CI 0.59–1.08, *p* = 0.07)	NCT00855218
SUN1170	3	First-line	Sunitinib vs. sorafenib	No difference in OS (HR 1.30, 95% CI 1.13–1.50, *p* = 0.999)	NCT00699374
BRISK FL	3	First-line	Brivanib vs. sorafenib	No difference in OS (HR 1.06, 95% CI 0.93–1.22, n.s.)	NCT00858871
BRISK PS	3	Second-line	Brivanib vs. placebo	No difference in OS (HR 0.89, 95% CI 0.69–1.15, *p* = 0.33)	NCT00825955
REACH	3	Second-line	Ramucirumab plus BSC vs. BSC	No difference in OS (HR 0.87, 95% CI 0.72–1.05, *p* = 0.14)	NCT01140347
EVOLVE-1	3	Second-line	Everolimus vs. placebo	No difference in OS (HR 1.05, 95% CI 0.86–1.27, *p* = 0.68)	NCT01035229
*Ongoing Trials*					
CheckMate-459	3	First-line	Nivolumab vs. sorafenib	*Recruiting*	NCT02576509
PHOCUS	3	First-line	Sorafenib +/− Pexa-Vec	*Recruiting*	NCT02562755
KEYNOTE-240	3	Second-line	Pembrolizumab vs. placebo	*Recruiting*	NCT02702401
RELIVE	3	Second-line	Doxorubin-TD vs. BSC	*Recruiting*	NCT01655693
REACH-2	3	Second-line	Ramucirumab vs. placebo	*Recruiting*	NCT02435433
METIV	3	Second-line	Tivantinib vs. placebo	*Recruitment closed*	NCT01755767

Displayed are selected clinical trials for first- and second-line systemic treatment in HCC. Abbreviations: OS, overall survival; TTP, time to progression; DEB-TACE, drug-eluting beads transarterial chemoembolization; TD, transdrug; BSC, best standard of care; HR, hazard ratio; CI, confidence interval; n.s., not significant.

**Table 2:** Assessment of target lesion response: Conventional RECIST vs. mRECIST for HCC following the AASLD-JNCI guideline [25].

Response	RECIST	mRECIST
CR	Disappearance of all target lesions	Disappearance of any intratumoral arterial enhancement in all target lesions
PR	At least a 30% decrease in the sum of diameters of target lesions, taking as reference the baseline sum of the diameters of target lesions	At least a 30% decrease in the sum of diameters of viable (enhancement in the arterial phase) target lesions, taking as reference the baseline sum of the diameters of target lesions
SD	Any case that does not qualify for either PR or PD	Any case that does not qualify for either PR or PD
PD	An increase of at least 20% in the sum of the diameters of target lesions, taking as reference the smallest sum of the diameters of target lesions recorded since treatment started	An increase of at least 20% in the sum of the diameters of viable (enhancing) target lesions, taking as reference the smallest sum of the diameters of target lesions recorded since treatment started

Abbreviations: AASLD, American Association for the Study of Liver Diseases; JNCI, Journal of the National Cancer Institute; HCC, hepatocellular carcinoma; mRECIST, modified Response Evaluation Criteria in Solid Tumors; CR, complete response; PR, partial response; SD, stable disease; PD, progressive disease.
